# Cotton Ascorbate Oxidase Promotes Cell Growth in Cultured Tobacco Bright Yellow-2 Cells through Generation of Apoplast Oxidation

**DOI:** 10.3390/ijms18071346

**Published:** 2017-06-23

**Authors:** Rong Li, Shan Xin, Chengcheng Tao, Xiang Jin, Hongbin Li

**Affiliations:** 1 College of Life Sciences, Key laboratory of Agrobiotechnology, Shihezi University, Shihezi 832003, China; lirongshzu@126.com (R.L.); cynthiaxs1102@hotmail.com (S.X.); taocc_124@163.com (C.T.); 2 Institute of Tropical Biosciences and Biotechnology, Chinese Academy of Tropical Agricultural Sciences, Haikou 571101, China; jinxianghainan@163.com

**Keywords:** *Gossypium hirsutum*, ascorbate oxidase, apoplast oxidation, cell elongation, auxin

## Abstract

Ascorbate oxidase (AO) plays an important role in cell growth through the modulation of reduction/oxidation (redox) control of the apoplast. Here, a cotton (*Gossypium hirsutum*) apoplastic ascorbate oxidase gene (*GhAO1*) was obtained from fast elongating fiber tissues. GhAO1 belongs to the multicopper oxidase (MCO) family and includes a signal peptide and several transmembrane regions. Analyses of quantitative real-time polymerase chain reaction (QRT-PCR) and enzyme activity showed that *GhAO1* was expressed abundantly in 15-day post-anthesis (dpa) wild-type (WT) fibers in comparison with *fuzzless-lintless* (*fl*) mutant ovules. Subcellular distribution analysis in onion cells demonstrated that GhAO1 is localized in the cell wall. In transgenic tobacco bright yellow-2 (BY-2) cells with ectopic overexpression of *GhAO1*, the enhancement of cell growth with 1.52-fold increase in length versus controls was indicated, as well as the enrichment of both total ascorbate in whole-cells and dehydroascorbate acid (DHA) in apoplasts. In addition, promoted activities of AO and monodehydroascorbate reductase (MDAR) in apoplasts and dehydroascorbate reductase (DHAR) in whole-cells were displayed in transgenic tobacco BY-2 cells. Accumulation of H_2_O_2_, and influenced expressions of Ca^2+^ channel genes with the activation of *NtMPK9* and *NtCPK5* and the suppression of *NtTPC1B* were also demonstrated in transgenic tobacco BY-2 cells. Finally, significant induced expression of the tobacco *NtAO* gene in WT BY-2 cells under indole-3-acetic acid (IAA) treatment appeared; however, the sensitivity of the *NtAO* gene expression to IAA disappeared in transgenic BY-2 cells, revealing that the regulated expression of the *AO* gene is under the control of IAA. Taken together, these results provide evidence that *GhAO1* plays an important role in fiber cell elongation and may promote cell growth by generating the oxidation of apoplasts, via the auxin-mediated signaling pathway.

## 1. Introduction

Cotton fibers derived from the seed coat as single epidermal cells are the most important materials in the textile industry. Fiber length and strength are the two most important parameters of fiber quality, determined by the overlapping process of fast elongation and secondary wall deposition [1]. Owing to its exceptional cell length, cotton fiber provides an excellent model material to study plant cell elongation development [2,3]. Cell elongation or enlargement is a crucial process in plant growth and morphogenesis. The plant hormone auxin and its mediated regulators, including reactive oxygen species (ROS), perform important roles in plant cell elongation and enlargement [4,5].

The apoplast comprises all of the external space outside of the plasmalemma including the cell wall and has a crucial role in the control of plant cell growth through regulating the reduction/oxidation (redox) balance of the cell. In apoplasts, ascorbate (Asc) is the unique small antioxidant molecule responsible for controlling the redox signaling and associating with ROS directly [6], and it exists in two forms including ascorbic acid (AsA) (reduced form) and dehydroascorbic acid (DHA) (oxidative form). DHA generated by apoplastic ascorbate oxidase (AO) was the key oxidative component that determines the redox status of the apoplast, thereby performing a decisive function in auxin signal transduction from the outside to the inside of the cell. Accumulated DHA resulted in the inhibition of auxin signal transmission by affecting the alteration of the membrane channel regulated by apoplastic transmembrane protein auxin-binding protein (ABP1) [6,7].

Ascorbate oxidase (AO) is the only key enzyme to produce the oxidative molecule DHA in the apoplast, and plays a significant role in cell elongation and enlargement development with extracellular localization and high activity in rapidly-expanding tissues [8,9,10]. In *AO*-overexpressing tobacco plants, the shoot elongation was dominantly promoted with a 3.5-fold enrichment of apoplastic DHA concentration and a 40-fold increase of AO enzyme activity [6]. The promotion or suppression of stem cell growth was observed in *AO* sense- or antisense-expressing tobacco plants [11]. The function of *AO* is inseparable from auxin; *AO* expression at the mRNA level was regulated by auxin in pumpkins [8], and AO can cause a change of auxin receptor sensitivity through the regulation of the oxidation of apoplasts, and, thus, influences auxin signal transduction [6,7].

Previously, we obtained the promoter sequence of the cotton ascorbate oxidase gene (*GhAO1*) and studied the promoter function in tobacco (*Nicotiana benthamiana*) plants [12]. In this work, we investigate the important role of the apoplastic *GhAO1* gene on cell growth in cultured tobacco bright yellow-2 (BY-2) cells. GhAO1 protein was localized in the cell wall and *GhAO1* was dominantly expressed in fiber elongating stages both at mRNA and protein levels. In *GhAO1*-overexpressing cultured tobacco BY-2 cells, cell growth was significantly promoted with accumulations of DHA and H_2_O_2_ in the apoplast, as well as an increase in apoplastic AO and monodehydroascorbate reductase (MDAR) enzyme activities. *NtAO* expression was enhanced or suppressed in wild type (WT) or *GhAO1*-overexpressing BY-2 cells under indole-3-acetic acid (IAA) treatment, respectively. We conclude that *GhAO1* may participate in fiber cell development by involvement in the auxin-mediated signaling pathway.

## 2. Results

### 2.1. Identification of Cotton Ascorbate Oxidase

**Figure 1:**
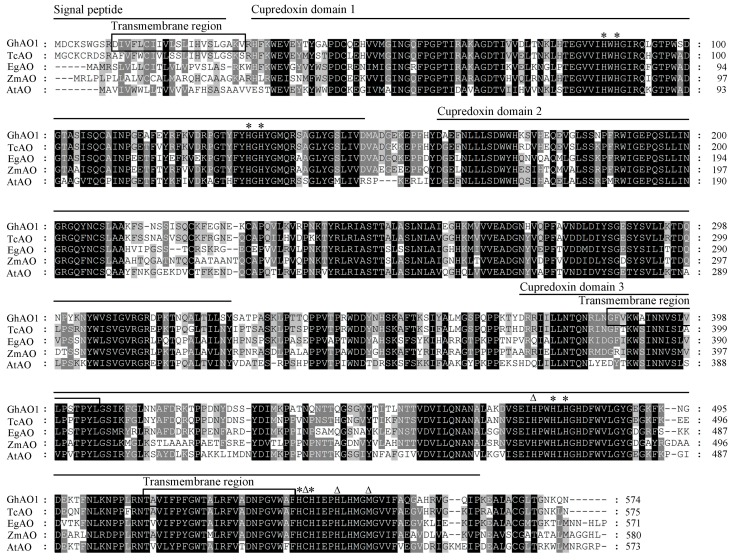
Amino acid sequence alignment of cotton (*Gossypium hirsutum*) ascorbate oxidase (GhAO1) protein. GhAO1 (Genbank accession no. KT794559) and four other plant ascorbate oxidase (AO) proteins of TcAO (Genbank accession no. XP_007016684.1), EgAO (Genbank accession no. XP_010925442.1), ZmAO (Genbank accession no. NP_001141087.1), and AtAO (Genbank accession no. NP_680176.5) were used for sequence alignment. Black shading indicates strictly-conserved residues whereas gray shading presents regions of less-strict conservation. Signal peptides, transmembrane regions, and three cupredoxin domains are indicated. Asterisks (*) indicate trinuclear copper binding sites and triangles (Δ) present Type 1 (T1) copper binding sites.

We obtained the ascorbate oxidase gene *GhAO1* (GenBank accession number: KT794559) from fast elongating fiber tissues by RT-PCR. The *GhAO1* full-length cDNA contained a 1716-bp open reading frame (ORF) and encoded a protein of 571 amino acid residues with a predicted molecular weight (*M*w) of 62.8 kDa. As shown in Figure 1, in alignments with homologous sequences from species including *Theobroma cacao*, *Elaeis guineensis*, *Zea mays*, and *Arabidopsis thalina*, the deduced amino acid sequence of GhAO1 displayed conservative characteristics of functional domains and belonged to the multicopper oxidase (MCO) family. A predicted signal peptide of 27 amino acid residues at the N-terminus and three transmembrane regions were found, implying that GhAO1 is a secreted extracellular protein. GhAO1 included three typical functional cupredoxin domain repeats distributed at residues of 44–161, 176–336 and 399–565, respectively, which are separated by several amino acid link sequences. In addition, three conserved-feature residue patterns, including two trinuclear copper binding sites characterized by conserved H–H–H–H copper ligands (asterisks) and one Type 1 (T1) copper binding site characterized by conserved H–C–H–M copper ligands (triangles), were discovered. These data provide the possibility that GhAO1 may function in ascorbate oxidation as a transmembrane extracellular protein using copper as a ligand.

### 2.2. Cotton (Gossypium hirsutum) Ascorbate Oxidase Gene (GhAO1) Is Preferentially Expressed during the Fiber Cell Fast Elongation Period at the mRNA Level and Total Protein Activity

**Figure 2:**
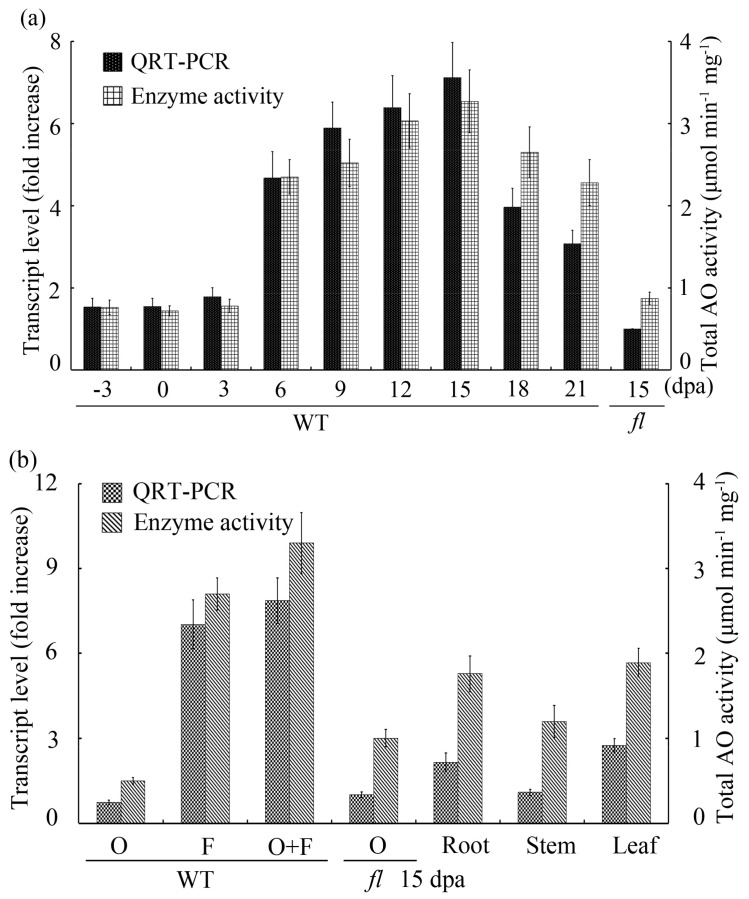
Expression pattern analysis of *GhAO1* during cotton fiber development stages. (**a**) Analyses of transcript and enzyme activity indicate that *GhAO1* is preferentially expressed in fast elongating fiber tissues. Wild-type cotton ovules associated with fibers at −3, 0, 6, 9, 12, 15, 18, 21 dpa and 10 dpa *fuzzless-lintless* (*fl*) mutant ovules were used for total RNA extraction and quantitative real-time polymerase chain reaction (QRT-PCR) analysis. QRT-PCR data of 10 dpa *fl* was artificially set to 1; (**b**) Tissue-specific analysis of *GhAO1* in different cotton materials. Different tissues of cotton plants, including ovules (O), fibers (F), and ovules associated with fibers (O+F) of 15 dpa wild type (WT), and 15 dpa *fl* mutant ovules, as well as roots, stems, and leaves, were used for QRT-PCR and enzyme activity analysis. The cotton ubiquitin gene *GhUBQ7* (Genbank accession no. AY189972) was included as the template control. Enzyme activity was determined by assaying the ascorbate oxidation photometrically and monitoring the absorbance at 265 nm using protein samples extracted from tissues of the different cotton plants presented. Error bars indicate the standard error from three independent experiments.

The expression patterns of GhAO1 in different fiber developmental stages were examined by quantitative real-time polymerase chain reaction (QRT-PCR) (Figure 2a). GhAO1 mRNA increased ~7-fold in 15-day post-anthesis (dpa) (the point of fastest fiber cell elongation) wild-type (WT) cotton ovules associated with fibers compared with 15 dpa fl mutant ovules, displaying that GhAO1 was particularly accumulated during fiber elongation development. The total AO activity showed the highest value at 15 dpa, which is consistent with the transcriptional level of the gene (Figure 2a). To further detect the tissue-specific feature of GhAO1, different cotton tissues including roots, leaves, stems, 15 dpa ovules and fibers were used for QRT-PCR and enzyme activity analysis. QRT-PCR analysis using RNA samples extracted from various tissues revealed that GhAO1 was abundantly expressed in WT fiber tissue, whereas relatively low levels of transcripts were observed in all of the other tissues. AO activity was measured through determining the oxidation rate of ascorbate. The total AO activities indicated a similar expression pattern with QRT-PCR data (Figure 2b). These results suggest that the expression of GhAO1 is specifically upregulated during cotton fiber elongation development.

### 2.3. GhAO1 Is a Cell Wall Protein

**Figure 3:**
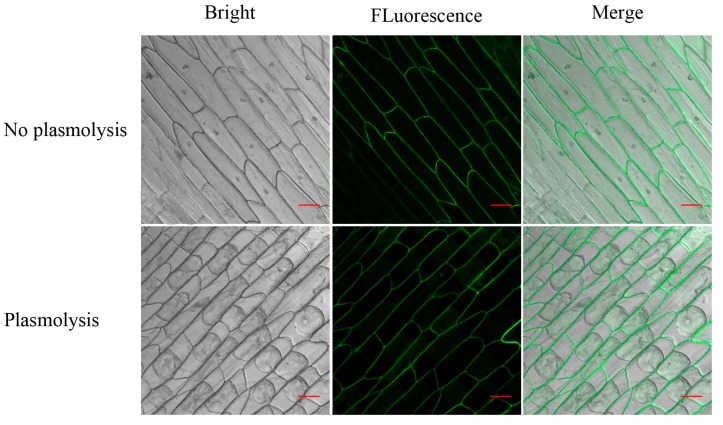
Subcellular localization of the GhAO1 protein in onion cells. Onion cells were transformed with *GhAO1::GFP* via the agrobacterium-mediated method. Mannitol was used to induce plasmolysis. Images are shown under bright, fluorescence, and merge conditions are indicated by confocal microscopy. Bar: 100 μm.

The subcellular distribution of GhAO1 was examined to further elucidate the regulation mechanism. The *GhAO1* gene was cloned into a modified pCAMBIA 2300-GFP vector to generate the *35S::GhAO1-GFP* construct. The fusion construct was driven by the cauliflower mosaic virus (CaMV) 35S promoter and ectopic overexpression was performed by transforming them into the onion epidermal cells using the agrobacterium-mediated method. After a subculture for 24 h, fluorescence microscopy visualizations of *GhAO1::GFP* displayed that the green fluorescent protein (GFP) signals overlapped in the extracellular space following detection by laser confocal imaging microscopy. Successive plasmolysis experiments of the transgenic onion cells were performed to verify, in-depth, the GhAO1 localization, which indicated that GFP green fluorescence were observed in the cell wall (Figure 3). The results supply a further confirmation that GhAO1 is a cell wall protein and may exert its biological function in the apoplastic space of the cell.

### 2.4. Overexpression of GhAO1 Promotes Cell Growth in Tobacco Bright Yellow-2 (BY-2) Cells

**Figure 4:**
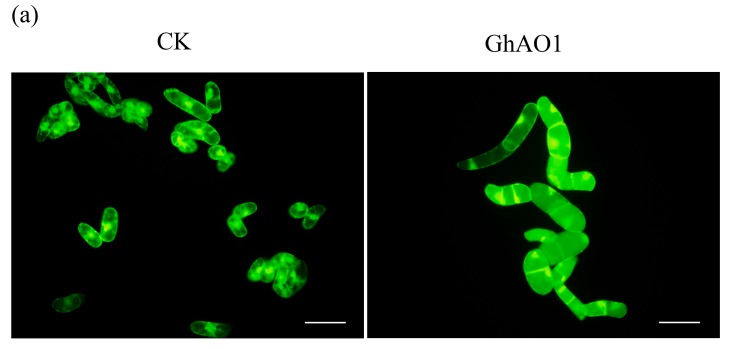

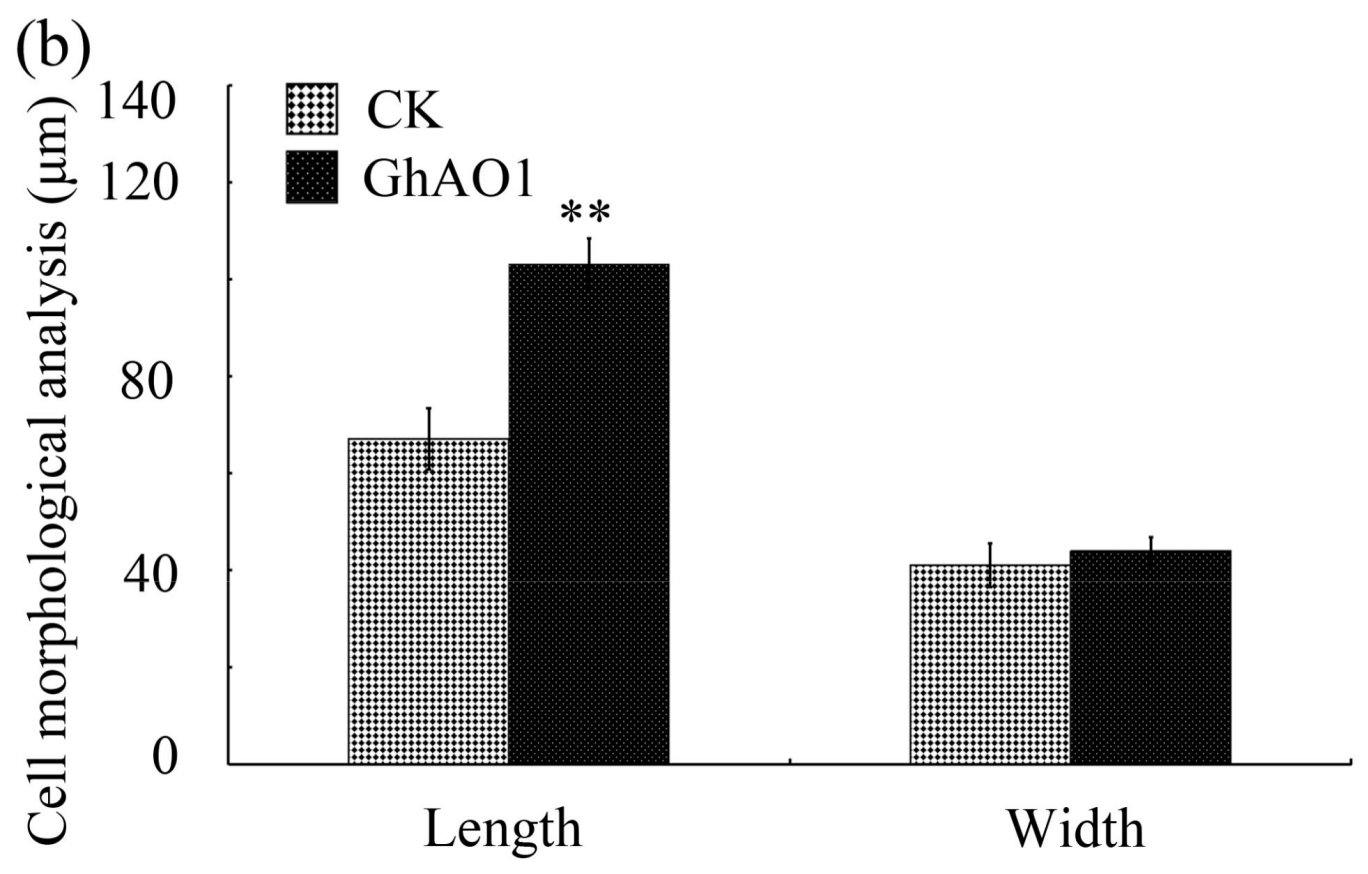
Cell morphological change of *GhAO1*-overexpressing transgenic BY-2 cells. (**a**) Representative cell morphology of transgenic BY-2 cells with ectopic overexpression of the vector and *GhAO1*. Images are shown under a fluorescence microscope. Bar: 100 μm; (**b**) Statistics of cell length and width. CK, transgenic cultured tobacco BY-2 cells overexpressing vector; GhAO1, transgenic tobacco BY-2 cells overexpressing *35S::GhAO1*; each value was the average of 100 mature BY-2 cells selected randomly in triplicate, independently. Error bars indicate the standard error from three independent experiments. Asterisks indicate significant differences according to Student’s *t* tests between CK and *GhAO1* transgenic BY-2 cells at the *p* < 0.01 level.

Cultured tobacco BY-2 cells were utilized to ascertain the correlation between *GhAO1* and cell growth. Among a set of generated *35S::GhAO1* BY-2 cell overexpression lines through the agrobacterium-mediated transformation method, overexpression lines with *GhAO1*-stable expression at transcription and protein activity levels were selected for successive detection, and the cells transformed with the vector were used as the control. Subculture transgenic cell lines were observed after staining with fluorescein diacetate (FDA) as well as a morphological test of cell length and width. Morphological observation and statistic data presented that the *GhAO1* transgenic cells were significantly promoted with a ~1.52-fold increase in length compared with the control cells (Figure 4), demonstrating that *GhAO1* is able to induce cell elongation growth predominantly.

### 2.5. DHA Is Enriched in GhAO1-Overexpressing Tobacco BY-2 Cells

**Figure 5:**
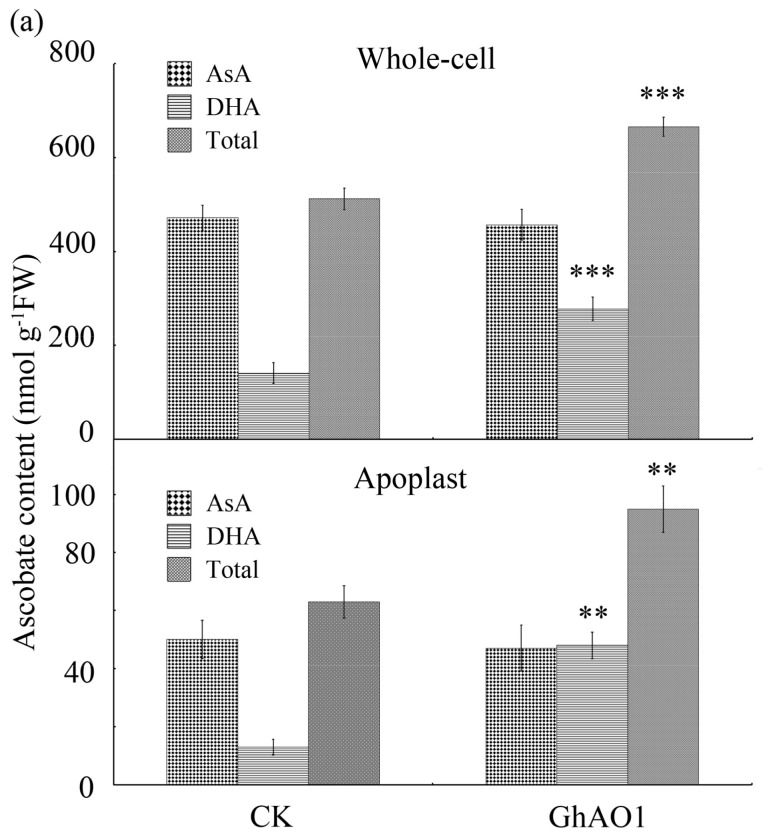

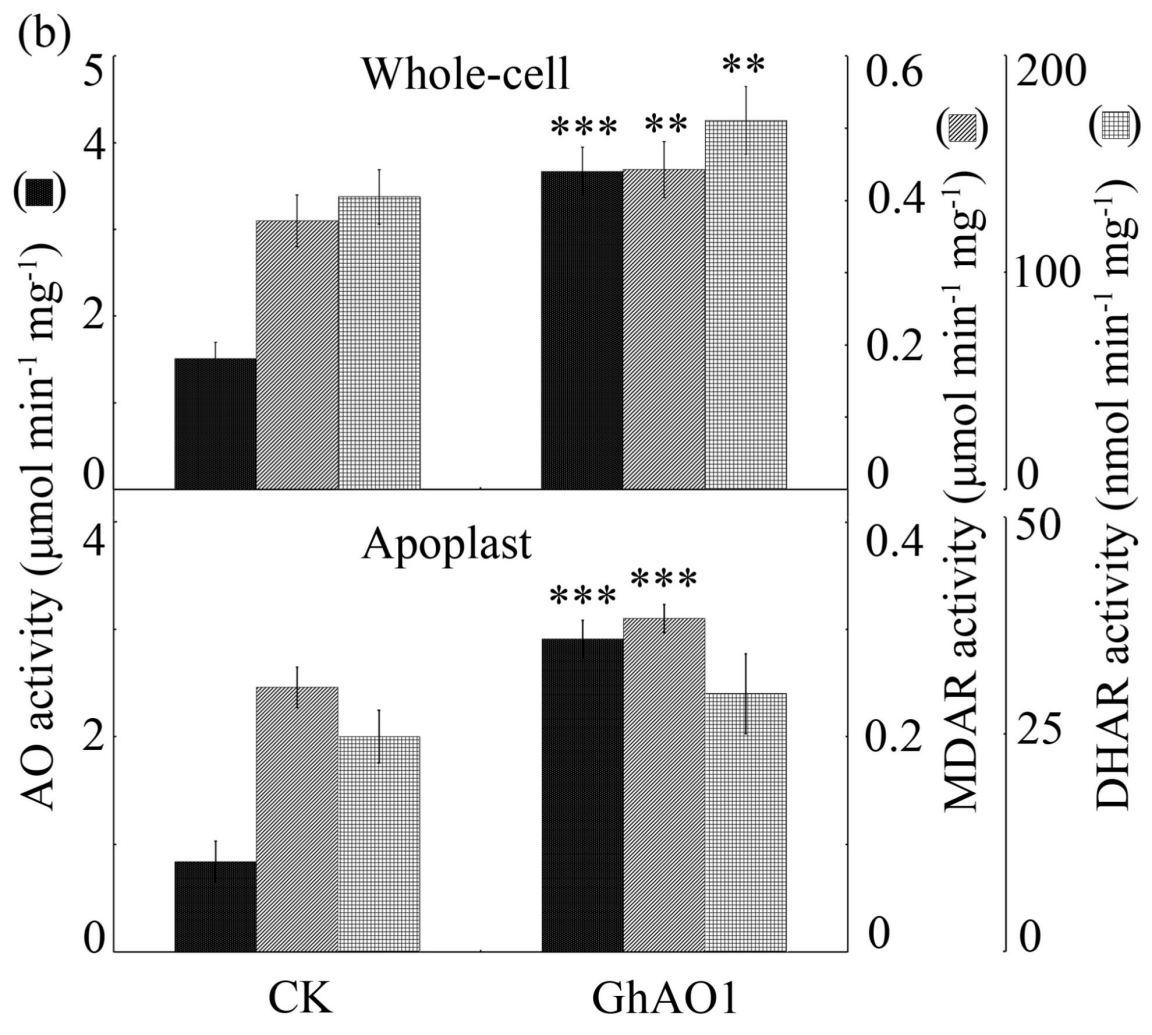
Changes in the levels of ascorbate acid (AsA) and dehydroascorbate acid (DHA) content and related metabolic enzyme activity in transgenic BY-2 cells. (**a**) Levels of AsA and DHA in whole-cell and apoplastic spaces of CK and *GhAO1* transgenic BY-2 cells; (**b**) Activity changes of AO, MDAR, and DHAR in whole-cell and apoplastic spaces of CK and *GhAO1* transgenic BY-2 cells. CK, transgenic cultured tobacco BY-2 cells overexpressing vector; GhAO1, transgenic tobacco BY-2 cells overexpressing *35S::GhAO1*; AO, ascorbate oxidase; MDAR, monodehydroascorbate reductase; DHAR, dehydroascorbate reductase. Values show the average of three independent measurements. Asterisks indicate significant differences according to Student’s *t* tests between CK and GhAO1, ** *p* < 0.01; *** *p* < 0.001.

Contents of total Asc, AsA, and DHA, as well as enzyme activities of AO, dehydroascorbate reductase (DHAR), and MDAR were measured to verify the relationship between *AO* expression and alterations of Asc different oxidation/reduction situations. In comparison of WT tobacco cells, *GhAO1*-overexpressing tobacco BY-2 cells indicated increased content of total Asc and DHA significantly in both the whole-cell and apoplastic space of the cell (Figure 5a). Meanwhile, in overexpressing tobacco BY-2 cells, AO and MDAR activities were enhanced in both the apoplast and whole-cell, and enzyme activity of DHAR, which is responsible for AsA recycling of the cell, showed a boosted expression in the whole-cell (Figure 5b). The results indicate that the induced generation of DHA through the AO catalyzing oxidation of AsA may be involved in the cell growth of transgenic tobacco BY-2 cells overexpressing *GhAO1*.

### 2.6. Ascorbate Oxidase (AO) Induces H_2_O_2_ Accumulation

**Figure 6:**
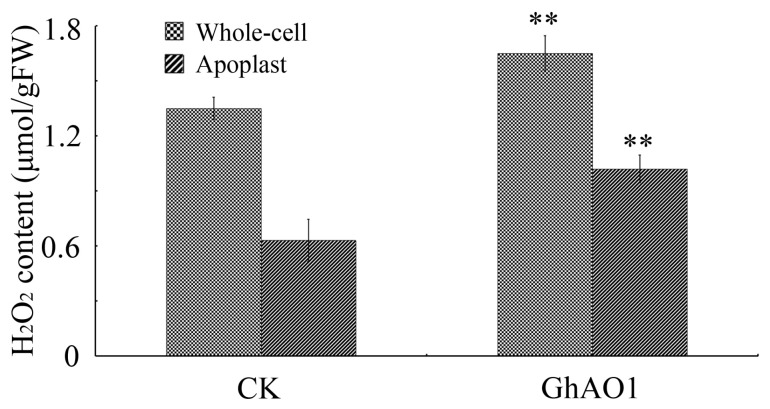
H_2_O_2_ is accumulated in the apoplastic space of transgenic BY-2 cells. H_2_O_2_ contents were determined in different cell compartments of whole-cells, apoplasts, and cytosols of transgenic tobacco BY-2 cells overexpressing vector or *35S::GhAO1*. Each value indicates the average of three independent measurements. CK, cultured tobacco BY-2 cells overexpressing vector; GhAO1, transgenic tobacco BY-2 cells overexpressing *35S::GhAO1*. Asterisks show significant differences according to Student’s *t* test between CK and GhAO1 at the *p* < 0.01 level.

In light of the crucial role played by H_2_O_2_ in cell elongation, we investigated the change of H_2_O_2_ content in transgenic BY-2 cells. H_2_O_2_ content was determined in different cell compartments of the whole-cell and apoplast. The result showed that H_2_O_2_ was substantially accumulated in whole-cells and apoplasts of transgenic tobacco BY-2 cells (Figure 6).

### 2.7. AO Affects Expression of Ca^2+^ Channel Genes

**Figure 7:**
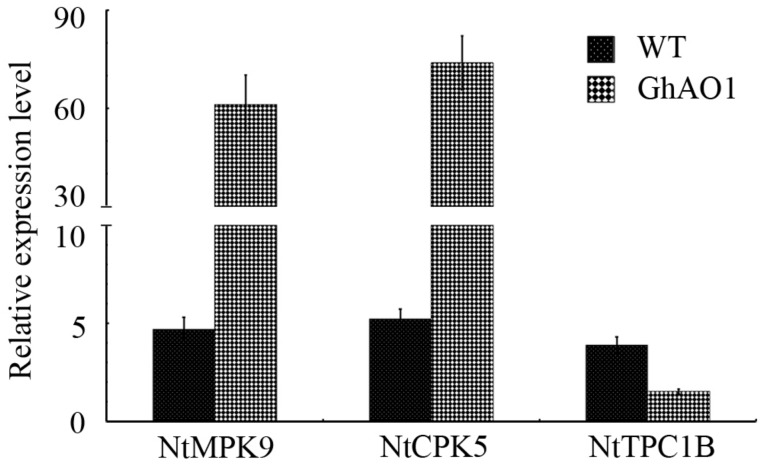
Expression analysis of Ca^2+^ channel genes in transgenic tobacco BY-2 cells. Transcript abundance was measured by QRT-PCR using total RNA extracted from materials of WT and transgenic tobacco BY-2 cells overexpressing *GhAO1* as template. Relative expression levels of *NtMPK9*, *NtCPK5*, and *NtTPC1B* were included, and the tobacco gene *18S rRNA* (Genbank accession no. AJ236016) was used as an internal control. Error bars indicate the standard error from three independent experiments.

In view of the critical role performed by calcium-mediated signal transduction in plant cell growth, the transcript expression levels of Ca^2+^ channel genes containing *NtMPK9*, *NtCPK5*, and *NtTPC1B* were measured in WT and transgenic tobacco BY-2 cells though QRT-PCR. In transgenic tobacco BY-2 cells overexpressing *GhAO1*, the activation of *NtMPK9* and *NtCPK5* with over 13- and 14-fold increase was observed, respectively, while the expression of *NtTPC1B* was distinctly suppressed with a 60% decrease (Figure 7).

### 2.8. AO Expression Is Modulated by Auxin

**Figure 8:**
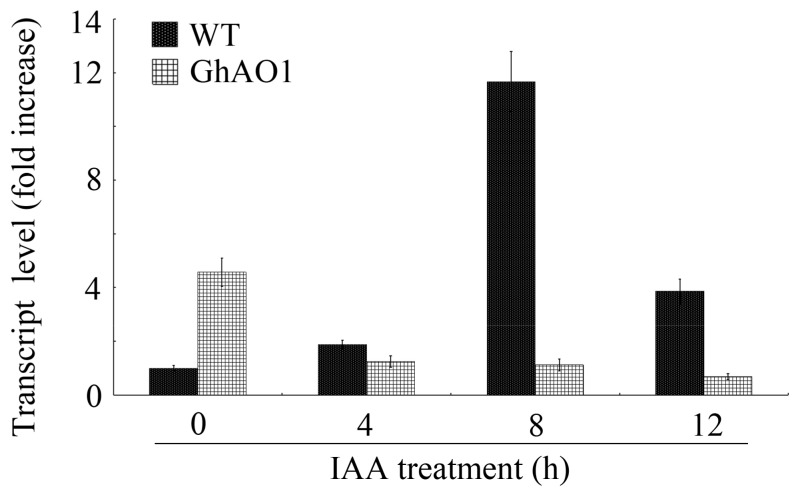
Tobacco *NtAO* gene expression analysis in wild-type (WT) and *GhAO1* transgenic BY-2 cells under indole-3-acetic acid (IAA) treatment. Materials of WT and transgenic BY-2 cells indicated were used for total RNA extraction and QRT-PCR analysis. The tobacco gene *18S rRNA* was applied as an internal control. QRT-PCR data of untreated WT BY-2 cells was artificially set to 1. Error bars indicate the standard error from three independent experiments.

As reported previously, the insensitivity of *AO* to auxin treatment was observed in *AO*-overexpressed plants [13]. To test the hypothesis in the *GhAO1*-overexpressing tobacco BY-2 cells, we measured the expression of the *NtAO* gene by QRT-PCR using RNA samples extracted from cultured transgenic tobacco BY-2 cells under auxin treatment. The results showed that the induced expression of *NtAO* was displayed in WT tobacco cells after 4 h IAA treatment, however, in *GhAO1*-overexpressing tobacco cells, *NtAO* indicated suppressed expression when IAA exists and illustrated a notable decrease of sensitivity to IAA (Figure 8). The data suppose that *AO* expression was modulated under the control of auxin, therefore determining the auxin signal transduction from the outside to the inside of the cell.

## 3. Discussion

In the apoplast, oxidative molecules such as ROS and DHA are crucial for the promotion of cell growth and development through the modulation of the redox state of the cell. Apoplastic AsA, as the unique reduced small molecule in the extracellular space, is the decisive component to the redox buffering capacity of the apoplast [14], and has principal potential to exert a crucial influence on cellular redox signaling [6,15,16,17]. Apoplastic ascorbate oxidase is the key enzyme that affects cell growth through oxidizing AsA to produce DHA, and, thus, controls the redox ratio of the apoplast [18]. The study of *AO* is not widespread, and *AO* genes have been cloned and characterized in many plants, including cucumber, pumpkin, tobacco, melon [19,20,21,22], etc. In the present work, an apoplastic acsorbate oxidase gene *GhAO1* was obtained from fast elongating fiber tissues, and the GhAO1 protein contained several cupredoxin domains and copper binding sites (Figure 1), implying its potential function in cellular redox reactions. *GhAO1* was highly upregulated at transcription level and enzyme activity during fiber cell elongation stages (Figure 2), which suggests that there is a close nexus between *GhAO1* and cell growth. Some plant *AO*s showed similar patterns that express high activities in rapidly-expanding tissues [8,22,23,24].

The cell wall is the key component in the apoplast that decides plant cell mechanical strength, which determines cell shape morphogenesis. Extracellular enzymes perform important functions in cell growth via modulating the changes of the apoplast [6]. In plant cells, cell elongation or expansion is coupled with cell wall loosening and rearrangement [25]. GhAO1 possessed a signal peptide and three transmembrane regions (Figure 1), indicating that GhAO1 is a secreted extracellular protein. Subcellular localization analysis showed that GhAO1 is a cell wall protein (Figure 3), providing a conceivable role of GhAO1 that may affect cell development through leading some alteration of the cell wall in a direct or indirect way. The study indicated that AO, at least in part, regulates cell wall architecture through oxidizing ascorbate to DHA, causing cell wall loosening, and therefore leading to cell growth [6].

The tobacco BY-2 cell line is an excellent material for studying cell development [26,27]. Significant cell growth was observed in *GhAO1*-overexpressing tobacco BY-2 cells (Figure 4), demonstrating again the *AO*’s important role in cell elongation growth. Overexpression of a pumpkin *AO* in tobacco BY-2 cells showed an increase of cells expanding compared to controls [28].

In plants, oxidative burst is considered to be crucial for cell development. Extracelullar oxidative molecules of ROS and DHA are important regulators in cell division and expansion [6,29,30,31]. Ascorbate is the only antioxidative molecule and plays multifunctions as coenzymes and cell growth regulator [6]. DHA, as the oxidised form of ascorbate produced by the the key enzyme AO in the apoplast, may produce a similar effect as oxidative burst leading to the apoplastic oxidation state [6,16], and, therefore, promotes cell enlargement through inducing depolarization of the plasma membrane and enhancing cell wall loosening [32,33]. In this work, *GhAO1*-overexpressing tobacco BY-2 cells displayed significant accumulation of DHA (Figure 5a), as well as high level of AO activity (Figure 5b). This is in accordance with the report, that in *AO*-overexpressing plants, DHA is significantly enriched in the apoplast, resulting in enhanced cell growth and stem elongation [18,34].

It has been reported that ROS, for instance H_2_O_2_ and the hydroxyl radical, are involved in plant growth and development [35]. In light of fiber development, significant ROS accumulation is observed, with enriched H_2_O_2_, to promote fiber cell elongation [36,37]. In addition, a high content of ROS production is observed in the fiber growth process that is catalyzed by GhPOX1 [38], which causes a substantial effect on cell expansion or enlargement by causing cell wall loosening [39,40]. In this study, H_2_O_2_ is accumulated in the apoplast of *GhAO1*-overexpressing tobacco plants (Figure 6). This is in agreement with the results that increased or decreased H_2_O_2_ content was observed in *AO*-overexpressing or *AO*-suppressing tobacco plants, respectively [11], as well as the report that *AO* overexpression induced the enhancement of H_2_O_2_ concentration [9]. An ascorbate metabolite could induce H_2_O_2_ generation in the apoplast [41]. The study reported that DHA produced by AO-catalyzed ascorbate degradation in the apoplast generates H_2_O_2_ [42], and H_2_O_2_ can produce DHA through the reaction with ascorbate, implying that DHA and H_2_O_2_ may strengthen each other co-operatively. In *AO*-overexpressing tobacco plants, enhanced H_2_O_2_ content was observed coupled with the decline of H_2_O_2_-detoxifying enzyme ascorbate peroxidase (APX), providing a possible link between H_2_O_2_ increase and APX decrease [11,43].

ROS generated in non-photosynthetic tissues catalyzed by NADPH oxidase and cell wall-associated extracellular peroxidases, coupled with the activation of Ca^2+^ channel (Ca2C), has been reported to perform a vital role in plant cell growth as a representative signaling link [44,45,46]. Many proteins, such as calcium-dependent protein kinase (CPK), plasmalemma-localized two-pore Ca^2+^ channel-associated protein (NtTPC1B), and mitogen-activated protein kinase (MPK), are crucial sensors or regulators in Ca2C signal transduction [13,25,47]. In transgenic tobacco BY-2 cells overexpressing *GhAO1*, H_2_O_2_ enrichment, followed with significant activated expressions of *NtMPK9* and *NtCPK5* and inhibited expression of *NtTPC1B* were indicated (Figure 6 and Figure 7), suggesting that AO may act in similar role in producing oxidative burst, and AO-derived ROS generation influences the Ca2C-permeable channel and thus enhances cell growth. This is consistent with the result of induced expression of a Ca^2+^ channel gene in *AO*-overexpressing plants [13], and the report of suppressed expression of *NtTPC1B* performed by AO [48]. The similar results of ROS, Ca2C, and cell growth have also been investigated in *Arabidopsis* root hairs [49].

Auxin-mediated acidification in the apoplast activates oxalate oxidase, which induces H_2_O_2_ accumulation, therefore promoting cell growth [6]. In addition, auxin influences the orientation of the cortical microtubules that determines cell wall arrangement, and auxin-induced production of free radicals is considered to be the key factor to increase cell wall extension [4]. Auxin performs a vital role in fiber development [4,50,51] via regulating extracellular oxidative signals, and has a direct link with *AO* expression [8,48]. The tobacco *NtAO* gene displayed an induced expression under IAA treatment, whereas the overexpression of *GhAO1* leads to the insensitivity of the tobacco *NtAO* gene to auxin (Figure 8), demonstrating that cotton *GhAO1* expression is modulated under the control of auxin. This is consistent with the result that *AO* induces the inactivation of IAA through catalyzing the reaction of oxidative decarboxylation [52]. In all, our results indicate that *GhAO1* is involved in fiber cell elongation development, and may stimulate cell growth through generating oxidative molecules regulated by auxin-mediated signal transduction.

## 4. Materials and Methods

### 4.1. Plant Materials

Cotton plants were cultivated in the field under natural conditions. On the day of anthesis, blooming flowers were marked. Bolls of WT cotton were harvested at −3, 0, 3, 6, 9, 12, 15, 18 and 21 dpa, respectively. After the harvest, ovules were excised from the bolls, and fibers were scraped from the ovules. Bolls of *fl* mutant cotton ovules were collected at 15 dpa as the control. All materials were immediately frozen in liquid nitrogen and stored at −80 °C for further use.

### 4.2. Functional Sequence Analysis

Protein sequences were aligned using the ClustalX program version 2.1 (European Bioinformatics Institute, Cambridge, UK) with default settings. Online software of conserved domains (CD) at the National Center for Biotechnology Information (NCBI) website (https://www.ncbi.nlm.nih.gov/Structure/cdd/wrpsb.cgi) was used for functional motif analysis, and online software packages of SignalP and TMpred from the ExPASy website (http://www.expasy.org/) were used for analyses of signal peptide and transmembrane regions.

### 4.3. Vector Construction

To construct the overexpression of plasmid *35S::GhAO1-GFP*, the *GhAO1* cDNA was amplified by PCR with specific primers (listed in Table S1) containing *BamH* I and *Sal* I restriction endonuclease sites. After enzymatic digestion and ligation, the *GhAO1* ORF sequence was inserted upstream of *GFP* gene of the modified expression vector pCAMBIA2300-GFP (provided by Professor Xianzhong Huang) between *Bam*H I and *Sal* I sites in the sense orientation. The constructed overexpression vector was used for further analyses of subcellular localization and tobacco genetic transformation.

### 4.4. RNA Extraction and Quantitative Real-Time Polymerase Chain Reaction (QRT-PCR)

Total RNA was extracted from different tissues of cotton plants and tobacco BY-2 cells, and then was used for cDNA preparation through a reverse transcription reaction according to the supplier’s recommendations. The cDNA was utilized as a template for further PCR analysis. The QRT-PCR reactions were performed using SYBR Green real-time PCR master mix with specific primers for different genes. *18S rRNA* and *UBQ7* were used as internal controls to normalize each sample for variations in the amount of initial RNA. All reactions were performed in triplicate independently. The primers used for QRT-PCR are shown in Table S1.

### 4.5. Subcellular Localization Analysis

Onion epidermal cells were used to detect the GhAO1 protein subcellular distribution. A piece of leaf was peeled from fresh onion and the inside epidermal of the onion leaf was separated. After soaking in 75% ethanol for 10 min and washing 3–4 times with sterile water, the inside epidermis was cut and co-cultivated with *Agrobacterium tumefaciens* carrying the *35S::GhAO1-GFP* vector on 1/2 MS medium at 28 °C under darkness. After a subculturing for 24 h, a confocal laser-scanning microscope (Zeiss LSM510, Oberkochen, Germany) was used to detect the GFP signals with an activation wavelength of 488 nm.

### 4.6. Isolation of Apoplastic Fluid

Tobacco BY-2 cells (~0.5 g) were collected, washed in distilled water, and infiltrated with vacuum (−60 kPa) for 5 min at 4 °C in 10 mM sodium buffer (pH 6) containing 1.5% (*w*/*v*) polyvinypyrrolidone (PVP), 1 mM ethylene diamine tetraacetic acid (EDTA), and 0.5 mM phenylmethylsulfonyl fluoride [53]. Then the material was blotted dry and placed into a 15-mL tube with a small hole penetrated in the bottom, which was put in a larger tube. After centrifugation at 1000× *g* for 10 min at 4 °C, the apoplastic fluid was collected. Both whole-cell and apoplastic extraction were used for further determinations of Asc, enzyme activity, and H_2_O_2_ content.

### 4.7. Ascorbate Determination

Tobacco BY-2 suspension cells were centrifuged at 3500 rpm for 2 min at room temperature to remove excess medium, and the collected precipitate was suspended with the assay mixture for ascorbate determination. Total ascorbate (reduced ascorbate + dehydroascorbate) was measured according to the detection of dipyridyl-Fe^2+^ complex generated by the reduction of Fe^3+^ to Fe^2+^ with the addition of 5 mM dithiothreitol (DTT) through the spectrophotometric method [54]. Reduced ascorbate was determined in the same way without DTT supplementation, and the dehydroascorbate concentration was obtained through calculating the difference between the contents of total and reduced ascorbate. Ascorbate content was calculated by comparison with standard known concentrations.

### 4.8. Assays of Enzyme Activities

Different cotton tissues and tobacco BY-2 cells were collected and homogenized with two volumes of extraction buffer containing 40 mM potassium phosphate (pH 7.0) and 0.5 mM EDTA. The supernatant was used for enzyme assays after centrifugation at 15,000× *g* for 10 min at 4 °C. Ascorbate oxidase activity was determined by measuring the decrease in the absorbance at 265 nm due to AsA oxidation in the reaction mixture containing 0.15 mM AsA. Dehydroascorbate reductase (DHAR) and monodehydroascorbate reductase (MDAR) activities were determined with the addition of 1 mM DTT, 2% (*w*/*v*) polyvinyl pyrrolidone (PVP), and 0.1% (*v*/*v*) Triton X-100 in the reaction mixture. The DHAR activity was calculated as the amount of ascorbate (mmol) produced per min. The MDAR activity was evaluated as NADH (mmol) oxidized per min. Protein assays were performed in triplicate using bovine serum albumin as the standard via Bradford’s method [55].

### 4.9. Transformation of BY-2 Cells

The constructed *35S::GhAO1* vector was transferred into *Agrobacterium tumefaciens* (strain GV3101) through the electroporation method and used to transfect WT tobacco (*Nicotiana tabacum*) bright yellow 2 (BY-2) cells. BY-2 cells were preserved on Linsmaier and Skoog solid medium under dark conditions by subculturing every seven days and culturing at 28 °C in a rotary shaker at 200 rpm. The transformed BY-2 cell colonies were obtained after culturing on fresh selection plates containing kanamycin (100 mg/mL) for 3–4 weeks. Selected resistant transgenic BY-2 cell colonies were transferred to liquid selection medium, including kanamycin, to generate cell suspensions for the following analysis.

### 4.10. Observation of BY-2 Cells

Transgenic tobacco BY-2 cells overexpressing *GhAO1* or vector were cultured in liquid selection medium for 2–3 h, and then were stained by fluorescein diacetate (FDA) for 10 min under dark conditions. Stained liquid BY-2 cells (~0.1 mL) were placed on a slide, and approximately 100 matured stained cells were selected randomly for measurement using a fluorescence microscope (Olympus BH2, Tokyo, Japan) with three independent experiments.

### 4.11. Treatment of Tobacco BY-2 Cells

Tobacco BY-2 cell suspensions were treated with 1 mM IAA, and both IAA-induced cells and WT control cells were harvested for subsequent analysis.

### 4.12. H_2_O_2_ Measurement

H_2_O_2_ concentration was determined using the spectrometric method by detecting the hydroperoxide-titanium complex at the absorbance of 405 nm as described previously [36]. Whole-cell, isolated apoplastic fluid, and cytosolic soluble fraction were utilized for assaying H_2_O_2_ content in acetone mixture through forming the hydroperoxide-titanium complex after the addition of 20% TiCl_4_ (*v*/*v* in 11 M HCl) and NH_4_OH. H_2_O_2_ content was evaluated according to the standard curve with known H_2_O_2_ concentrations.

### 4.13. Accession Numbers

Sequence data used in the article were obtained from GenBank with accession numbers KT794559 (*GhAO1*), NM_001324873.1 (*NtMPK9*), FJ026805.1 (*NtCPK5*), AB124647 (*NtTPC1B*), AJ236016 (*18S rRNA*), and AY189972 (*GhUBQ7*).

## Supplementary Materials

Supplementary materials can be found at www.mdpi.com/1422-0067/18/7/1346/s1.
