# Supplementary material for: Cotton Ascorbate Oxidase Promotes Cell Growth in Cultured Tobacco Bright Yellow-2 Cells through Generation of Apoplast Oxidation
Source: Int J Mol Sci. 2017 Jun 23;18(7):1346. doi: 10.3390/ijms18071346 (PMC5535839; doi:10.3390/ijms18071346)
Supplement: Supplementary file 1 [file ijms-18-01346-s001.pdf]

Supplementary Materials: Cotton Ascorbate Oxidase Promotes Cell Growth in Cultured Tobacco Bright Yellow-2 Cells through Generation of Apoplast Oxidation

Rong Li, Shan Xin, Chengcheng Tao, Xiang Jin and Hongbin Li

Table S1. Primers used in the present study

Primer Name	Primer Sequence (Underlines Indicate the Restriction Sites of *Bam*H I and *Sal* I)
GhAO1F	5′-CGGGATCCATGGATTGTAAATCTTGGGGTT-3′
GhAO1R	5′-ACGCGTCGACGTTCTGCTTGTTTCCTGTTAAT-3′
GhUBQ7F	5′-CAGATCTTCGTCAAAACCCT-3′
GhUBQ7R	5′-GACTCCTTCTGGATGTTGTA-3′
GhAO1QRTF	5′-CATAGTGCCCGTGATAGA-3′
GhAO1QRTR	5′-CTTCATACCGAGGGAGTT-3′
NtAOF	5′-ATTTCATTCACGCTCCTCC-3′
NtAOR	5′-GTGGCATTCACATTCGGCT-3′
NtMPK9F	5′-TCGTTTCCCCTTACCCC-3′
NtMPK9R	5′-GGATGTGCGTTTGTGGC-3′
NtCPK5F	5′-CATCTTCCGACCAAACTA-3′
NtCPK5R	5′-TGGCGATTGACTTACACG-3′
NtTPC1BF	5′-CACCTTTGGCTGGCTGTAT-3′
NtTPC1BR	5′-TGGCAACAAAAGCACGAT-3′
18SF	5′-GGTGGAGCGATTTGTCTGGT-3′
18SR	5′-CAGGCTGAGGTCTCGTTCGT-3′
